# Acute kidney injury in pediatric burn patients

**DOI:** 10.1007/s00467-024-06341-5

**Published:** 2024-03-22

**Authors:** Demet Kahramanlar, Sare Gülfem Özlü, Pervin Demirci, Elif Emel Erten, Emrah Şenel, Umut Selda Bayrakçi

**Affiliations:** 1 Ankara Koru Hospital, Ankara, Turkey; 2 Department of Pediatric Nephrology, Faculty of Medicine, Ankara Bilkent City Hospital, Ankara Yıldırım Beyazıt University, University District 1604, Street No: 9, Çankaya, Ankara Turkey; 3 Department of Biostatistics, Faculty of Medicine, Ankara Yıldırım Beyazıt University, Ankara, Turkey; 4 Department of Pediatric Surgery, Ankara Bilkent City Hospital, Ankara, Turkey; 5 Department of Pediatric Surgery, Faculty of Medicine, Ankara Bilkent City Hospital, Ankara Yıldırım Beyazıt University, Ankara, Turkey

**Keywords:** Acute kidney injury, Burn, Pediatric, Hemoglobin

## Abstract

**Background:**

Acute kidney injury (AKI) is a common and important complication of burn injury. Although there are numerous adult studies, data regarding AKI in pediatric burn patients are scarce. Here, we aimed to evaluate the frequency, clinical features, and prognosis of AKI among pediatric burn injury patients.

**Methods:**

This is a retrospective cohort study. Patients aged between 1 month and 18 years who had been followed up between the years 2011 and 2017 were included, and patients with previous kidney disease were excluded. Demographic data, laboratory and clinical variables, management strategies, and outcome data were obtained from the hospital records. Factors associated with AKI were determined by logistic regression analysis.

**Results:**

A total of 697 patients had been followed up, and 87 (12.5%) had AKI. Older age, refugee status, prolonged duration between the incident and time of hospitalization, presence of sepsis, severity and type of burn, volume of fluid administration, intubation status, and accompanying organ failure were all associated with the development of AKI. According to multivariate logistic regression analysis, the most statistically significant factors associated with the development of AKI were older age and increased serum hemoglobin values. In terms of outcomes, length of stay and mortality increased in patients with AKI when compared with patients without AKI.

**Conclusion:**

Similar to adults, AKI is an important and common complication of burn injury in pediatric burn patients and is associated with increased length of stay, morbidity, and mortality. Early recognition and prompt and appropriate management are crucial to avoid morbidity and mortality.

**Graphical abstract:**

**Figure:**
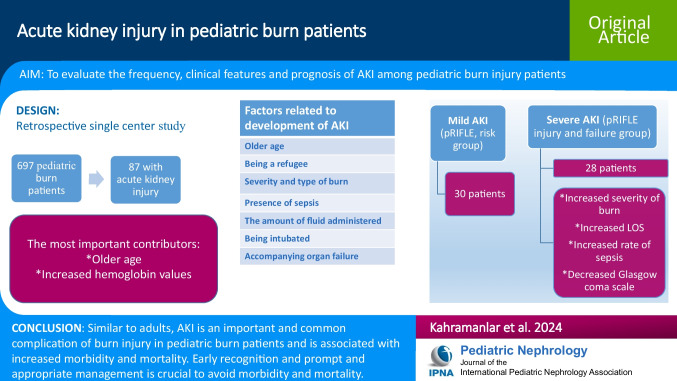
A higher resolution version of the Graphical abstract is available as Supplementary information

**Supplementary Information:**

The online version contains supplementary material available at 10.1007/s00467-024-06341-5.

## Introduction

Acute kidney injury (AKI) is a frequent and major complication of severe burns [1]. In previous studies, the incidence of AKI was reported to be between 1 and 64% in burn patients [2]. Recently, a systematic review including 33 studies and 8200 burn injury patients reported the incidence of AKI as 38% (30–46%) and the incidence of patients who received kidney replacement therapy as 12% (8–16%) [3]. These differences originated from diverse inclusion criteria and discrete definitions of AKI used in the studies [1].

Risk factors of AKI development are also evaluated in several adult reports; total body surface area of burn (TBSA), presence of sepsis, older age, and inhalation injury are demonstrated to be the most common risk factors of AKI [3–7]. It is already known that AKI not only increases the length of hospital stays and morbidity but also increases mortality in burn patients [1–4]. Recent reports also demonstrated that AKI increases the risk of chronic kidney disease in burn injury survivors [2–4, 8–10].

Although there are numerous studies evaluating the incidence, risk factors, and outcome of AKI in adult burn patients, data regarding the pediatric age group are scarce [3]. The aim of this study was to evaluate the frequency and risk factors of AKI and determine the outcome of AKI in burn patients who had been followed up in our pediatric burn intensive care unit (ICU).

## Patients and methods

We conducted a retrospective cohort study of pediatric patients who were admitted to our 16-bed specialized pediatric burn ICU between 2011 and 2017. All patients admitted to the burn ICU, between the ages of 1 month and 18 years, were included in the study. Patients with known previous kidney disease (acute and/or chronic kidney disease) were excluded. Demographic data included age, gender, race, and the duration between the burn incident and admission to the pediatric burn ICU. Clinical features such as weight, height, body mass index (BMI), TBSA, mean blood pressure, urine output, degree of burn, severity of burn, type of burn injury (milk, water, other fluids; flame, electrical burn, inhalation, chemical), presence of sepsis, intubation, Glasgow Coma Scale, and type of fluid administered at admission were evaluated. Fluid resuscitation was performed according to the Parkland formula [11]. Laboratory parameters such as kidney function tests as well as eGFR, blood counts, inflammatory markers, and liver function tests were collected from hospital records. Median length of hospital stay (LOS) and mortality of the patients with AKI were also evaluated.

This study was approved by the University of Health Sciences, Ankara Child Health and Diseases Hematology Oncology Training, and Research Hospital Ethics Committee (date: 20.03.2018, approval number: 2018/026). The study was conducted in accordance with the principles of the Declaration of Helsinki.

### Definitions

TBSA was calculated according to Lund and Browder’s diagram [12].

The severity of burn was classified according to the National Burns Treatment Algorithm published by Yastı et al. [13] which combined the degree of burn and TBSA as:

(i) *Minor burns*: Second-degree child burns less than 10% TBSA or third-degree child or adult burns less than 2% TBSA
(ii) *Moderate burns*: Second-degree child burns involving 10 to 20% TBSA or third-degree child or adult burns involving 2 to 10% TBSA
(iii) *Major burns*: Second-degree burns greater than 20% of TBSA or third-degree adult or child burns greater than 10% TBSA

Sepsis was diagnosed according to the current criteria [14].

### Definition of AKI

Patients with elevated creatinine at admission according to the age-related values were defined as having AKI when the basal creatinine values were not available [15]. Early AKI was defined as AKI occurring in the first 3 days of hospitalization. AKI was defined according to pediatric RIFLE criteria in patients whose required data for classification could be accessed appropriately (the lowest score for eGFR or urine output) [16].

We evaluated the effects of the demographic variables (gender, age, ethnicity, time of admission), clinical features (presence of sepsis, degree of burn, TBSA, severity of burn, intubation, Glasgow Coma Scale, mean arterial pressure, accompanying organ failure, and the type and amount of fluid administered), and laboratory parameters (urea, creatinine, sodium, potassium, chloride, calcium, aspartate aminotransferase, alanine aminotransferase, hemoglobin, white blood cell, platelets, CRP) on the development of early AKI.

We also compared the demographic data, clinical variables, and laboratory features according to the pediatric RIFLE classifications at the time of hospitalization when data were available. The risk group of pRIFLE was defined as mild AKI, and the injury and failure group was defined as severe AKI. Demographic data, clinical variables, and laboratory features were compared.

### Outcome

Demographic, clinical, and laboratory parameters that affected outcome and mortality were evaluated. The effect of AKI on mortality was also investigated.

### Statistical methods

The conformity of the numerical variables examined within the scope of the study to the normal distribution was evaluated graphically and with the Shapiro-Wilks test. The median (minimum; maximum) was used to summarize the numerical variables, and mean ± standard deviation values were given as additional information. Categorical variables were summarized by frequency (*n*) and percentage.

In comparison between categorical variables, the appropriate method results from Pearson’s, Fisher’s exact chi-squared, and Yates-corrected chi-squared tests were given. The Bonferroni-corrected results in multiple comparisons were summarized. In case the number of pRIFLE groups was insufficient on arrival, the results of the Monte Carlo–corrected chi-squared test were given. The Mann–Whitney *U* test was used for comparisons of two independent groups, and the Kruskal–Wallis test was used for comparisons of more than two groups. In case of significant difference, the Dunn Bonferroni–corrected results were given.

Univariate logistic regression analysis was performed for the variables whose effects on clinically predicted AKI at admission, odds ratio, and 95% confidence intervals were given. As a result of univariate analysis, the results of the multivariate logistic regression model made with the Forward:Wald method were given, considering the variables with *p* < 0.25 and clinically recommended to be in the model. The Hosmer and Lemeshow test results were used to evaluate the model’s goodness of fit, and the test result was not found to be significant (*p* < 0.05), indicating that the model had a good fit with the data. The Box-Tidwell approach was used to examine whether numerical variables were linearly related to logit. If necessary, numeric variables were divided into two classes based on the median value. Statistical analyses were performed with IBM SPSS Statistics for Windows, Version 21.0 (IBM Corp. Released 2012, Armonk, NY). Statistical significance level was accepted as *p* < 0.05.

## Results

Between 2011 and 2017, a total of 697 patients aged between 1 month and 18 years were admitted to the Pediatric Burn ICU. Among them 427 (61%) were male, and the mean age of the patients was 36 months (minimum 1 month, maximum 216 months). Six hundred and thirteen of the patients (88%) were of Turkish origin, and the remaining 84 (12%) were refugees.

Eighty-seven patients (12.5%) had AKI at admission or within 72 h of admission.

### Factors affecting AKI

A total of 60 (69%) of the 87 patients with AKI and 367 (60.2%) of the patients without AKI were boys (*p* = 0.115). Patients who had AKI were older than patients without AKI (*p* < 0.001). Refugees made up 19.5% of the patients with AKI and 11% of those without (*p* = 0.034).

Older age, being a refugee, prolonged duration between the incident and time of hospitalization, presence of sepsis, severity and type of burn, the amount of fluid administered, being intubated, and accompanying organ failure all affected the development of AKI (Table 1).

**Table 1:** Factors affecting AKI at admission

	Total (*n* = 697)	AKI at admission		*Z*, *χ*^2^; *p*
		Non-AKI (*n* = 610)	AKI (*n* = 87)	
Gender				
Male	427 (61.3)	367 (60.2)	60 (69.0)	*Z* = 2.485; 0.115
Female	270 (38.7)	243 (39.8)	27 (31.0)	
Age (month)				
Median (min; max)	36 (0.5; 216)	36 (0.5; 216)	60 (0.5; 204)	*Z* = − 4.627; < **0.001**
Mean ± SD	56.94 ± 54.15	53.16 ± 51.49	83.50 ± 64.37	
Nationality				
Residents	613 (87.9)	543 (89.0)	70 (80.5)	*χ*^2^ = 4.483; **0.034**
Refugee	84 (12.1)	67 (11.0)	17 (19.5)	
Admission time				
Median (min; max)	6 (1; 720)	6 (1; 700)	14 (1; 720)	*Z* = − 4.362; < **0.001**
Mean ± SD	22.73 ± 69.53	19.66 ± 60.82	44.29 ± 111.33	
Sepsis				
No	549 (78.8)	497 (81.5)	52 (59.8)	*χ*^2^ = 20.169; < **0.001**
Yes	148 (21.2)	113 (18.5)	35 (40.2)	
Degree of burn				
First	177 (25.4)	161 (26.4)	16 (18.4)	*χ*^2^ = 4.914; 0.178
Second superficial + deep	340 (48.7)	299 (49.0)	41 (47.1)	
Third	158 (22.7)	132 (21.6)	26 (29.9)	
Fourth	22 (3.2)	18 (3.0)	4 (4.6)	
Percentage of burn				
Median (min; max)	18 (3; 90)	17 (3; 80)	25 (5; 90)	*Z* = − 4.287; < **0.001**
Mean ± SD	21.52 ± 13.57	20.46 ± 12.40	29.01 ± 18.37	
Severity of burn				
Mild	214 (30.7)	195 (32.0)	19 (21.8)	*χ*^2^ = 8.869; **0.012**
Moderate	200 (28.7)	180 (29.5)	20 (23.0)	
Severe	283 (40.6)	235 (38.5)^a^	49 (55.2)^b^	
Etiology of burn				
Milk	61 (8.8)	51 (8.4)	10 (11.5)	*χ*^2^ = 11.631; **0.040**
Water	352 (50.5)	320 (52.5)^a^	32 (36.8)^b^	
Flame	173 (24.8)	143 (23.4)^a^	30 (34.5)^b^	
Other	57 (8.2)	49 (8.0)	8 (9.2)	
Electrical	39 (5.6)	32 (5.2)	7 (8.0)	
Other (ınhalational + contact + chemical)	15 (2.2)	15 (2.5)	0 (0.0)	
Glasgow Coma Scale (at admission)				
Median (min; max)	15 (1; 15)	15 (1; 15)	15 (5; 15)	*Z* = − 4.897; < **0.001**
Mean ± SD	14.05 ± 2.48	14.24 ± 2.18	12.69 ± 3.72	
Intubation				
Yes	42 (6.0)	26 (4.3)	16 (18.4)	*χ*^2^ = 24.403; < **0.001**
No	655 (94.0)	584 (95.7)	71 (81.6)	
Accompanying organ failure				
No	608 (87.2)	543 (89.0)	65 (74.7)	*χ*^2^ = 12.731; < **0.001**
Yes	89 (12.8)	67 (11.0)	22 (25.3)	
Mean blood pressure		*n* = 79	*n* = 13	
Median (min; max)	110.70 (67.42; 157.83)	110.67 (67.42; 149.87)	113.68 (100.54; 157.83)	*Z* = − 1.833; 0.067
Mean ± SD	110.89 ± 16.39	109.50 ± 16.00	119.33 ± 16.87	
Amount of fluid administered (*n* = 119) (ml)		*n* = 110	*n* = 9	
Median (min; max)	1500 (190; 9000)	1467.5 (190; 9000)	1820 (1300; 7150)	*Z* = − 2.315; **0.021**
Mean ± SD	1741.18 ± 1187.57	1637.18 ± 996.05	3012.22 ± 2306.22	

Among laboratory parameters, urea, creatinine, AST, ALT, and hemoglobin values were significantly increased (*p* < 0.05), and calcium and thrombocyte levels were significantly decreased among patients with AKI (*p* < 0.05) (Table 1).

When univariate logistic regression analysis was performed, it was observed that AKI risk was 1.9 times greater among refugee patients (*p* = 0.024). The presence of sepsis increased the risk of AKI by 2.96 times (*p* < 0.001). The risk of AKI increased 2.5 times in those with a median age of over 36 months compared to those under 36 months of age (*p* = 0.001). While the degree of burn did not increase the risk of AKI at admission, an increase of 10 units in the percentage of burns increased the risk of AKI by 1.4 times (*p* < 0.001). The risk of AKI increased 2 times in patients with major burns when compared with minor burns (*p* = 0.010).

According to univariate logistic regression analysis, mean blood pressure, amount of fluid administrated, and levels of CRP, leukocytes, and thrombocytes did not affect the presence of AKI at admission (*p* > 0.005). AKI risk increased 2 times among patients who had a hemoglobin level above 12.8 g/dl when compared to the patients with a hemoglobin level under 12.8 g/dl (*p* = 0.003) (Table 2).

**Table 2:** Factors affecting AKI according to univariate logistic regression analysis

Variables	Univariate logistic regression analysis			
	OR	95% CI for OR	Wald	*p*
Gender (F/M)	1.471	0.908–2.383	2.464	0.117
Age (≤ 36/ > 36 months)	2.509	1.576–3.993	15.047	** < 0.001**
Nationality (residents/refugees)	1.968	1.094–3.542	5.102	**0.024**
Admission time (≤ 6/ > 6 h)	2.330	1.456–3.728	7.560	**0.006**
Sepsis (no/yes)	2.960	1.842–4.759	20.079	** < 0.001**
Degree of burn				
First			4.821	0.185
Second superficial/deep	1.380	0.751–2.536	1.075	0.300
Third	1.982	1.020–3.850	4.079	0.043
Fourth	2.236	0.674–7.417	1.730	0.188
Percentage of burn (for every 10 units)	1.038 (1.448)	1.024–1.053 (1.262–1.661)	26.919	** < 0.001**
Severity of burn				
Mild			8.639	**0.013**
Moderate	1.140	0.590–2.206	0.152	0.696
Severe	2.096	1.192–3.685	6.613	**0.010**
Glasgow score (≥ 15/ < 15)	2.866	1.785–4.604	18.973	** < 0.001**
Intubation (no/yes)	5.062	2.591–9.889	22.525	** < 0.001**
Accompanying organ failure (no/yes)	2.743	1.589–4.735	13.120	** < 0.001**
Mean blood pressure (≤ 110.70/ > 110.70)	2.428	0.690–8.539	1.910	0.167
Amount of fluid administered (≤ 1500 cc/ > 1500 cc)	3.500	0.830–14.762	2.910	0.088
CRP (≤ 1.7/ > 1.7)	1.214	0.774–1.905	0.713	0.399
Leukocyte (≤ 17.5/ > 17.5)	0.984	0.628–1.542	0.005	0.943
Thrombocyte (PLT) (≥ 376/ < 376)	1.576	0.998–2.489	3.807	0.051
Hemoglobin (HGB) (≤ 12.8/ > 12.8)	2.046	1.283–3.264	9.045	**0.003**

*OR* odds ratio, *CI* confidence interval

As a result of the multivariate logistic regression analysis performed with the prospective addition method using the variables that are clinically predicted to influence AKI, age, time of admission, TBSA, intubation, and hemoglobin values were found to be significant (Table 3). When other variables in the model were kept constant, the risk of developing AKI in those older than 36 months was 1.885 (1.9 times) times higher than those ≤ 36 months (*p* = 0.012).

**Table 3:** Variables affecting AKI at admission (multivariate logistic regression analysis)

Variables	Multivariate logistic regression analysis			
	OR	95% CI for OR	Wald	*p*
Age (≤ 36/ > 36 months)	1.885	1.151–3.086	6.346	**0.012**
Time of admission (≤ 6/ > 6 h)	1.847	1.129–3.022	5.963	**0.015**
Percentage of burn (every 10 units)	1.026 (1.297)	1.010–1.043 (1.109–1.517)	9.817	**0.002**
Intubation (no/yes)	2.508	1.163–5.410	5.499	**0.019**
Hemoglobin (HGB) (≤ 12.8/ > 12.8)	1.773	1.07–2.916	5.086	**0.024**
Constant	0.026		124.241	** < 0.001**

Method: the Forward:Wald, Hosmer, and Lemeshow tests *p* = 0.805; rate of correct classification 88.4

### Demographic features and clinical variable pRIFLE classes

Among 87 patients who developed AKI, pRIFLE classification could be performed in 58 patients for whom required data was obtained. We divided the patients into mild AKI based on the “risk” category of pRIFLE and severe AKI based on the “injury and failure’’ category of pRIFLE. Thirty patients were in the mild AKI group, and 28 patients were in the severe AKI group. Table 4 includes the comparison of the demographic and clinical variables of the patients regarding their pRIFLE group status. It is demonstrated that sepsis risk is increased in the “injury” group (*p* = 0.043) and that sepsis was significantly more common in the severe AKI group (*p* = 0.036). Also, the severity of burn significantly increased the severity of AKI (*p* = 0.007). Glasgow Coma Scale was lower in the severe AKI group (*p* = 0.036). The other features did not differ significantly between the AKI groups (Table 4).

**Table 4:** Clinical variables according to RIFLE classification

	RIFLE (risk: mild; injury-failure: severe)		*Z*, Monte Carlo *χ*^2^; *p*
	Mild (*n* = 30)	Severe (*n* = 28)	
Gender			
Male	22 (73.3)	19 (67.9)	*χ*^2^ = 0.210; 0.647
Female	8 (26.7)	9 (32.1)	
Age (month)			
Median (min; max)	90 (12; 192)	51 (12; 192)	*Z* = 1.735; 0.083
Mean ± SD	104.53 ± 71.98	69.00 ± 49.57	
Nationality			
Local residents	27 (90.0)	22 (78.6)	Fisher *p* = 0.290
Refugees	3 (10.0)	6 (21.4)	
Time of admission			
Median (min; max)	18.5 (1; 480)	9 (1; 294)	*Z* = 0.203; 0.839
Mean ± SD	40.33 ± 99.86	27.43 ± 57.50	
Length of stay			
Median (min; max)	12 (2; 68)	17.5 (1; 101)	*Z* = 2.097; **0.036**
Mean ± SD	14.10 ± 14.48	21.07 ± 19.14	
Sepsis			
No	22 (73.3)	13 (46.4)	*χ*^2^ = 4.381; **0.036**
Yes	8 (26.7)	15 (53.6)	
Degree of burn			
First	11 (36.7)	2 (7.1)	*χ*^2^ = 8.120; 0.044
Second (superficial, deep)	11 (36.7)	15 (53.6)	
Third	6 (20.0)	10 (35.7)	
Fourth	2 (6.7)	1 (3.6)	
Percentage of burn			
Median (min; max)	19 (5; 70)	30 (10; 80)	*Z* = 2.268;0.023
Mean ± SD	23.7 ± 15.54	33.25 ± 18.43	
Severity of burn			
Mild	13 (43.3)	2 (7.1)	*χ*^2^ = 9.902; **0.007**
Moderate	5 (16.7)	8 (28.6)	
Severe	12 (40.0)	18 (64.3)	
Etiology of burn			
Milk	1 (3.3)	7 (25.0)	*χ*^2^ = 5.918; 0.205
Water	11 (36.7)	7 (25.0)	
Flame	13 (43.3)	10 (35.7)	
Other liquids	2 (6.7)	2 (7.1)	
Electricity	3 (10.0)	2 (7.1)	
Glasgow Coma Scale (at admission)			
Median (min; max)	15 (5; 15)	14 (5; 15)	*Z* = 2.080; **0.038**
Mean ± SD	13.43 ± 3.43	11.75 ± 4.18	
Intubation (at admission)			
Intubated	5 (16.7)	7 (25.0)	*χ*^2^ = 0.613; 0.434
Not intubated	25 (83.3)	21 (75.0)	
Accompanying organ failure			
No	24 (80.0)	17 (60.7)	*χ*^2^ = 2.600; 0.107
Yes	6 (20.0)	11 (39.3)	

*Z*, Mann–Whitney *U* test; *χ*^2^ = the appropriate chi-squared test statistic determined based on the distribution of groups

### Outcomes

In terms of outcomes, median length of stay and mortality increased in patients with AKI when compared to patients without AKI. Median length of hospitalization stay (LOS) was 12 days for patients with early AKI (min, 1 day; max, 101 days) and 9 days for the patients without early AKI (min, 1 day; max, 123 days). Median LOS significantly increased among patients with AKI at admission (*Z* =  − 2.990; *p* = 0.003). Also, median LOS was increased with increased severity of AKI (*p* = 0.036) (Table 4).

Sixteen of the patients (18.4%) who had AKI at admission and 5 (0.8%) of the patients who did not have AKI at admission died during ICU follow-up (Table 5). Among the patients who were discharged from the hospital, 71 (10.5%) had AKI (*p* < 0.001). Other factors affecting mortality are summarized in Table 5.

**Table 5:** Factors affecting mortality in pediatric burn patients

	Total (*n* = 697)	Alive (*n* = 676)	Deceased (*n* = 21)	*Z*, *χ*^2^; *p*
Gender				
Male	427 (61.3)	413 (61.1)	14 (66.7)	*χ*^**2**^** = **0.083; 0.773
Female	270 (38.7)	263 (38.9)	7 (33.3)	
Age				
Median (min; max)	36 (0.5; 216)	36 (0.5; 216)	84 (12; 192)	*Z* = − 2.792; **0.005**
Mean ± SD	56.94 ± 54.15	55.91 ± 53.66	90.29 ± 60.67	
Ethnicity				
Turk	613 (87.9)	601 (88.9)	12 (57.1)	Fisher *p* < **0.001**
Refugee	84 (12.1)	75 (11.1)	9 (42.9)	
Admission time				
Median (min; max)	6 (1; 720)	6 (1; 700)	24 (2; 718)	*Z* = − 4.747; < **0.001**
Mean ± SD	22.73 ± 69.53	20.89 ± 62.73	82.0 ± 177.86	
Sepsis				
No	549 (78.8)	546 (80.8)	3 (14.3)	Fisher *p* < **0.001**
Yes	148 (21.2)	130 (19.2)	18 (85.7)	
Degree of burn				
First	177 (25.4)	177 (26.2)^a^	0 (0.0)^b^	*χ*^**2**^** = **17.219; **0.001**
Second superficial/deep	340 (48.7)	332 (49.1)	8 (38.1)	
Third	158 (22.7)	147 (21.7)^a^	11 (52.4)^b^	
Fourth	22 (3.2)	20 (3.0)	2 (9.5)	
Percentage of burn				
Median (min; max)	18 (3; 90)	18 (3; 80)	50 (13; 90)	Z = − 6.487; < **0.001**
Mean ± SD	21.52 ± 13.57	20.65 ± 12.47	49.57 ± 17.72	
Degree of burn				
Mild	214 (30.7)	214 (31.7)^a^	0 (0.0)^b^	*χ*^**2**^** = **26.888; < **0.001**
Moderate	200 (28.7)	199 (29.4)^a^	1 (4.8)^b^	
Severe	283 (40.6)	263 (38.9)^a^	20 (95.2)^b^	
Etiology of burn				
Milk	61 (8.8)	57 (8.4)	4 (19.0)	*χ*^**2**^** = **14.508; **0.013**
Water	352 (50.5)	348 (51.5)^a^	4 (19.0)^b^	
Flame	173 (24.8)	164 (24.3)	9 (42.9)	
Other liquids	57 (8.2)	53 (7.8)	4 (19.0)	
Electrical injury	39 (5.6)	39 (5.8)	0 (0.0)	
Other (inhalational + contact + chemical)	15 (2.2)	15 (2.2)	0 (0.0)	
Glasgow Coma Scale (at admission)				
Median (min; max)	15 (1; 15)	15 (1; 15)	12 (5; 15)	*Z* = − 5.692**; < 0.001**
Mean ± SD	14.05 ± 2.48	14.16 ± 2.31	10.38 ± 4.49	
Intubation (at admission)				
Yes	42 (6.0)	34 (5.0)	8 (38.1)	Fisher *p* < **0.001**
No	655 (94.0)	642 (95.0)	13 (61.9)	
Accompanying organ failure				
No	608 (87.2)	599 (88.6)	9 (42.9)	Fisher *p* < **0.001**
Yes	89 (12.8)	77 (11.4)	12 (57.1)	
Mean blood pressure (*n* = 92)				
Median (min; max)	110.70 (67.42; 157.83)	110.7 (67.42; 157.83)	110.75 (84.48; 136.79)	*Z* = − 0.181; 0.856
Mean ± SD	110.89 ± 16.39	110.85 ± 16.29	111.48 ± 20.14	
Amount of fluid administered (*n* = 119)				
Median (min; max)	1500 (190; 9000)	1492.5 (190; 7150)	2000 (1820; 9000)	*Z* = − 2.012; **0.041**
Mean ± SD	1741.18 ± 1187.57	1675.69 ± 991.96	4273.3 ± 4094.40	
AKI at admission				
No	610 (87.5)	605 (89.5)	5 (23.8)	Fisher *p* < **0.001**
Yes	87 (12.5)	71 (10.5)	16 (76.2)	

*χ*^**2**^** = **the appropriate chi-squared test statistic determined based on the distribution of groups

*Min* minimum, *Max* maximum, *SD* standard deviation, *Z* Mann–Whitney *U* test

^a,^^b^There is a significant difference between column proportions indicated by different letters (*p* < 0.05)

## Discussion

As already reported in previous studies, AKI is a well-known complication of burn injury, and it has a significant impact on morbidity and mortality. Pediatric reports evaluating AKI in burn patients are relatively scarce, so in this study, we aimed to investigate the incidence, risk factors, and outcome of early AKI in pediatric burn patients. We detected the incidence of AKI as 12.3%, and this was much lower than previously reported studies [5]. Univariate logistic regression analysis revealed that older age, time of admission, TBSA, sepsis, severity and type of burn, being intubated, and accompanying organ failure were found to be significant clinical risk factors for AKI. According to multivariate logistic regression analysis, age, time of admission, and TBSA were the most important contributors to AKI. Multivariate logistic regression analysis also revealed that hemoglobin values were found to be the most important risk factor for AKI.

The incidence of AKI is reported to be between 1 and 64% in previous studies. In a recent meta-analysis by Wu et al., the authors reported the pooled incidence of AKI as 39.6% (between 22.5 and 65.5%) [7]. The studies in this meta-analysis were mainly composed of adult patients and commonly used RIFLE criteria for AKI classification [7]. In the study by Palmieri et al., the incidence of AKI was detected to be 45% in pediatric burn patients [17]. In this study, the authors also used RIFLE criteria for AKI classification [17]. We applied pRIFLE criteria when the required data were available; when data were not available, we used elevated creatinine levels for the definition of AKI. Like the results of Palmieri et al.’s study, more severe cases were observed in the “injury” group than the “risk” group [17]. Our AKI definition did not consider diuresis which likely underestimates the true number of AKI cases in our study. This different classification of AKI may explain the markedly lower incidence of AKI in our study. The comparison of the incidence of AKI between studies is difficult due to differences in the definition of AKI and inclusion criteria [17].

In adult studies, older age is reported to be a risk factor for AKI because older patients are much more prone to comorbidities such as diabetes mellitus, hypertension, or heart failure [1, 3, 5, 7, 18]. There are no specific data on the contribution of age to AKI from the previous pediatric studies [17]. In our study, the age of the patients with AKI was significantly older than the patients without AKI. We think that the effect of age is completely different from adult patients, and we could speculate that the effect of older age in pediatric patients on AKI could be due to the possibly riskier overall environment of older children.

An important finding in our study was the increased incidence of AKI among refugee patients. Previous reports have demonstrated that burn injury is one of the most common reasons for hospital admission after upper respiratory tract infections and gastroenteritis among refugees [19–21]. Authors have attributed the increased incidence of burns in their studies to the impact of low socioeconomic status, overcrowded living conditions, and open-floor cooking and heating [19–21]. They also indicated that these undesirable living conditions not only cause more burn injuries but also may cause more severe burns. As demonstrated in TBSA, we believe that increased burn severity may also increase the risk of AKI among refugees. In addition, difficulties in accessing healthcare facilities may prolong the time between burn event and hospitalization in this population, which may contribute to the development of AKI.

Fluid replacement has long been demonstrated as the cornerstone of standard care for burn injury [1, 4, 22]. Previous studies clearly indicate that appropriate fluid resuscitation is necessary to avoid hypovolemic shock, early AKI, and multiple organ dysfunction [1, 4, 7]. In a comprehensive study by Alobaidi et al., the authors demonstrated that fluid overload is common among critically ill patients and strongly associated with poor outcomes [23]. Although univariate logistic regression analysis did not demonstrate a clear association between fluid replacement and AKI, the amount of fluid administered was increased in AKI patients when compared to non-AKI patients. This increased amount of fluid administered to AKI patients was attributed to the more severe clinical course and increased demand for fluid in these patients. Due to the retrospective nature of our study, we were not able to determine the fluid overload in our patients accurately, but we must state that clinicians should also keep in mind fluid overload in burn victims.

While multivariate logistic regression analysis did not detect an independent association, sepsis was found to be an important risk factor for AKI according to univariate logistic regression analysis in our study. In fact, this is somewhat unexpected because previous studies demonstrated that sepsis is particularly related to late AKI [3, 5, 6, 17]. Considering that previous studies mostly consisted of adult patients, we speculated that the association of sepsis and early AKI in our pediatric cohort could be related to the fact that children are more prone to inflammation, and as a result, sepsis may also develop earlier than in adults. Another remarkable finding in our study was the relation between admission time and AKI. We detected that the prolonged time between the burn event and hospitalization increased the development of AKI. This could be attributed to the delay in fluid resuscitation. In addition, we could speculate that this time, lag may also have contributed to the development of early sepsis in our patients.

It has been reported that increased depth and size of burned areas are well-known risk factors for AKI in burn injury [1–3, 5–7, 16, 17, 24]. We found similar results in our study. We demonstrated that a 10-unit increase in burn percentage increases the risk of AKI by 1.4 times, and severe burns increase the risk of AKI by 2 times, compared to mild burns.

As mentioned, in previous studies, this is not surprising because increased TBSA and increased severity of burn are clear indications of disease burden in burn patients [1–7, 17, 18, 24]. TBSA is the main determinant of an individual’s physiological response and consequent organ failure [1].

Type of burn injury, particularly inhalation injury, has been identified as a clear risk factor for AKI [5, 18, 25]. In a recent meta-analysis by Wu et al., the authors reported that only flame injury was a risk factor for AKI, and this could be attributed to muscle necrosis and subsequent rhabdomyolysis caused by deep flame injury [7]. In our study, we could not detect the effect of inhalation injury or flame injury because of the small number of patients in these groups. Burn injury caused by various forms of liquids was related to AKI, but this again was not independently associated with AKI.

Intubation and mechanical ventilation are other important risk factors for AKI in burns [5]. In our study, multivariate logistic regression analysis revealed that intubation was an independent risk factor for AKI. In a comprehensive study by Ho et al., the authors pointed out that intubation reflects more severe disease and greater extent of burn, and once intubated, patients are susceptible to ventilator-associated pneumonia, catheter infections, and hypotension due to medications used to achieve sedation [5]. Besides the risk of intubation itself, all these factors could contribute to the development of AKI in burn victims [5].

Laboratory parameters were also evaluated as potential markers for AKI in our study. Increased hemoglobin values (over 12.8 g/dl) nearly doubled the risk of AKI (*p* = 0.003). This increased risk may be due to the hypovolemia secondary to burn injury. Previous studies also demonstrated that increased hemoglobin values and heme proteins can lead to acute kidney damage [26, 27]. In an interesting study by Karakaya et al. where they created burn injury criteria, hemoglobin values were detected as a factor that affects the development of AKI [27]. The authors suggest that in cases where hemoglobin levels do not decrease despite fluid resuscitation, phlebotomy may be applied to prevent acute kidney damage [27].

As clearly demonstrated in previous reports, AKI increases both in-hospital and outpatient morbidity and mortality in patients with burn injuries. In line with these studies, LOS and mortality were also increased in patients with AKI when compared with patients without AKI [1, 3, 5, 6, 17, 18, 25]. We also demonstrated that mortality rates are increased in the severe AKI group as reported in previous studies [28].

We are aware that there are some limitations in our study. The main limitation is the retrospective nature of the study that resulted in insufficient data collection for the classification of AKI. We were not able to evaluate the urinary output of our patients. We could not apply pRIFLE criteria to all of our patients, which could have led us to underestimate the incidence of AKI in our cohort.

Besides these limitations, we think that evaluating AKI in pediatric burn injury patients is valuable considering the limited number of studies in this age group. We think that our study will make a significant contribution to the literature in this area.

## Conclusions

AKI is an important complication of burn injury in pediatric patients as observed in adults. The most important risk factors for AKI are age, time between the burn incident and hospital admission, percentage of burn, and hemoglobin values. AKI significantly increases morbidity and mortality in pediatric burn patients. The most important point of management is early recognition and appropriate fluid resuscitation. Multicentric studies with large numbers of pediatric patients are required to characterize risk-stratification tools to prevent AKI and to develop standardized management strategies.

### Supplementary Information

Below is the link to the electronic supplementary material.

Graphical abstract (PPTX 92.3 KB)

## Data Availability

The dataset is available from the corresponding author on reasonable request.
