# Supplementary material for: Acute kidney injury in pediatric burn patients
Source: Pediatr Nephrol. 2024 Mar 22;39(8):2515–24. doi: 10.1007/s00467-024-06341-5 (PMC11199209; doi:10.1007/s00467-024-06341-5)
Supplement: Supplementary file 1 — Graphical abstract (PPTX 92.3 KB) [file 467_2024_6341_MOESM1_ESM.pptx]

## Slide 1
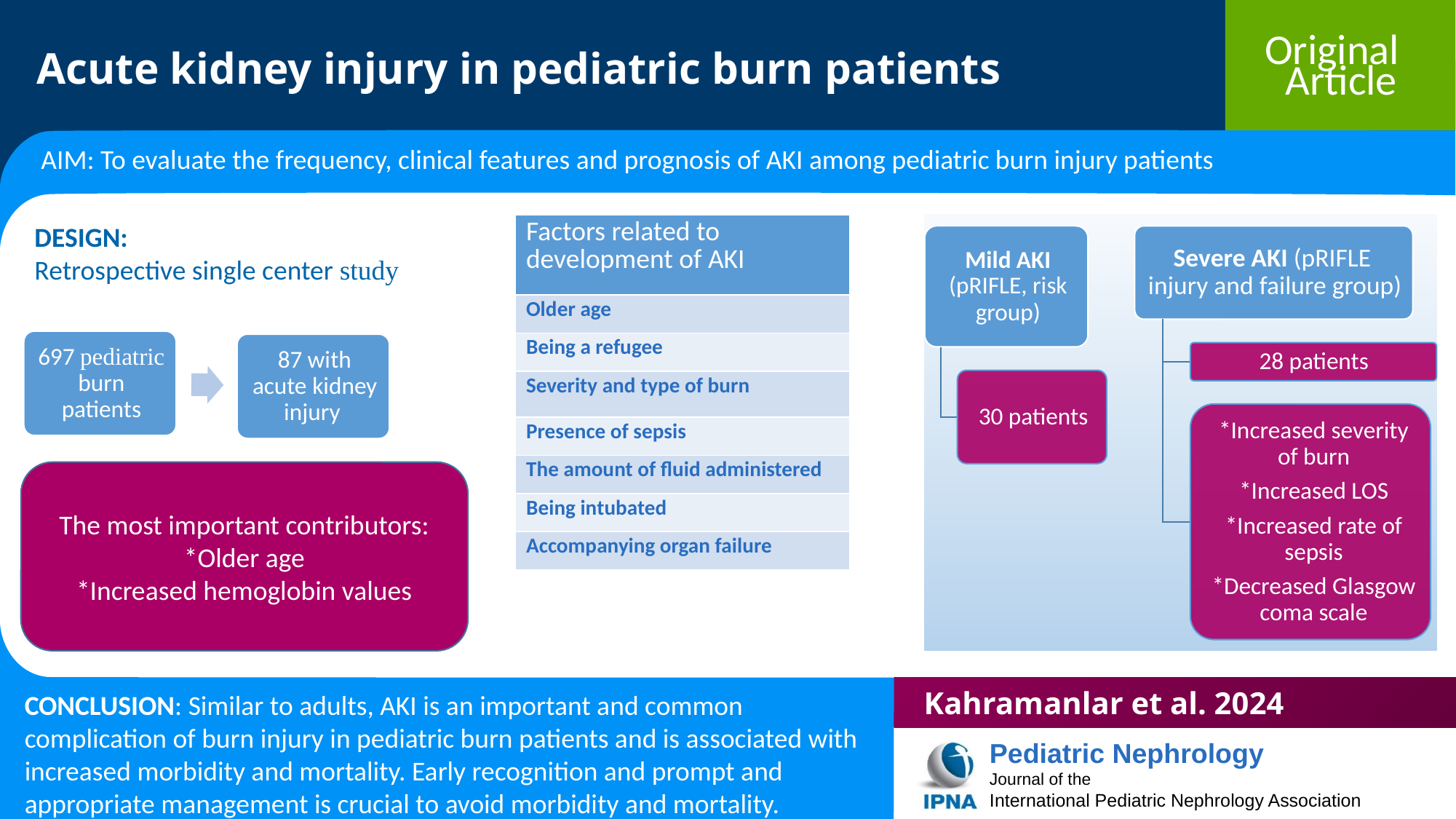

Acute kidney injury in pediatric burn patients
AIM: To evaluate the frequency, clinical features and prognosis of AKI among pediatric burn injury patients
DESIGN:
Retrospective single center study
| Factors related to development of AKI |
| --- |
| Older age |
| Being a refugee |
| Severity and type of burn |
| Presence of sepsis |
| The amount of fluid administered |
| Being intubated |
| Accompanying organ failure |
The most important contributors:
*Older age
*Increased hemoglobin values
Kahramanlar et al. 2024
CONCLUSION: Similar to adults, AKI is an important and common complication of burn injury in pediatric burn patients and is associated with increased morbidity and mortality. Early recognition and prompt and appropriate management is crucial to avoid morbidity and mortality.
